# Increased nucleotide metabolism alleviates Alzheimer’s disease pathology

**DOI:** 10.1038/s41419-025-08066-1

**Published:** 2025-10-16

**Authors:** Yizhou Yu, Michael B. Miller, August Yue Huang, Bryan Wei Zhi Tan, Ivana Celardo, Nuno Santos Leal, Samantha H. Y. Loh, L. Miguel Martins

**Affiliations:** 1 MRC Toxicology Unit, University of Cambridge, Gleeson Building, Cambridge, UK; 2 Healthspan Biotics Ltd, Milner Therapeutics Institute, Cambridge, UK; 3 Division of Neuropathology, Department of Pathology, Brigham and Women’s Hospital, Harvard Medical School, Boston, MA USA; 4 Division of Genetics and Genomics, Department of Paediatrics, Boston Children’s Hospital, Harvard Medical School, Boston, MA USA

**Keywords:** Alzheimer's disease, Alzheimer's disease, Experimental models of disease, Transcriptomics, Genetics research

## Abstract

Genetic information in cells flows from DNA to RNA to proteins, which form molecular machines. During normal ageing, cell intrinsic and environmental factors alter this flow of information by damaging DNA in cells, including postmitotic neurons. Damage to DNA is associated with age-related neurodegenerative diseases such as Alzheimer’s disease (AD). We previously reported an increase in DNA repair mechanisms in a fly model of AD. However, the causal mechanisms underlying somatic mutations in AD remain unclear. Here, we combine in silico methods from single-cell genomics of patients with AD with experimental validation in a *Drosophila* model of AD to elucidate the DNA repair processes in AD. We show that the levels of poly(ADP‒ribose) polymerase 1 (PARP1), which mediates multiple DNA damage repair pathways, are increased in the brains of patients with AD. We found that higher *PARP1* levels in neurons from patients with AD are linked to increased disease risk and a greater burden of somatic mutations. Nucleotide imbalance can increase the frequency of somatic mutations upon activation of DNA repair processes. Using a fly model of AD, we identified a metabolic signature in AD animals characterised by decreased levels of phosphorylated nucleotides. Enhancing nucleotide metabolism via dietary supplementation or genetic manipulation protects against AD pathology in animals. Finally, Mendelian randomisation revealed that higher expression of human deoxyguanosine kinase (DGUOK) is linked to a lower risk of developing AD. Our results suggest that enhancing nucleotide metabolism could improve DNA repair and serve as an adjunct therapy to delay AD progression.

## Introduction

Alzheimer’s disease (AD) is a neurodegenerative disorder characterised by progressive cognitive decline and dementia [1]. The accumulation of DNA damage is a well-established ageing factor and a critical pathological cause of AD (reviewed in [2]). Compared with non-AD individuals, neurons in AD brains accumulate more DNA changes, referred to as somatic mutations or somatic single-nucleotide variants (sSNVs), and these variants can lead to functional consequences in genomic regions crucial for neuronal function and survival [3].

Poly(ADP‒ribose) polymerase (PARP) activation is an early response that initiates the repair of damaged DNA [4]. The PARP family comprises 17 members involved in diverse processes, including DNA repair, transcription regulation, and chromatin remodelling. Among them, PARP1 is the most abundant and plays a key role in sensing DNA strand breaks and catalysing the addition of poly(ADP-ribose) chains to recruit components of the DNA repair machinery. Higher PARP activity has been hypothesised to be essential for maintaining genome stability across the lifespan of the organism [5]. Postmortem brain samples from patients with AD also have increased poly(ADP-ribosyl)ation [6]. Suppressing PARP genetically [7] and pharmacologically [8–10] has been shown to delay neurodegeneration in AD in cells, flies and mice. However, these treatments only moderately delay AD pathology. A leading hypothesis on the mechanism by which PARP overactivation leads to cell death is that it causes neurodegeneration by depleting NAD^+^, resulting in mitochondrial dysfunction [11, 12]. Although PARP suppression has been proposed as a potential therapeutic target [8, 9, 12], the endogenous role of PARP is unclear. It is possible that neurons respond to DNA damage by upregulating PARP1. Suppressing this cellular response might be detrimental to the ability of cells to repair damaged DNA and exacerbate the load of unrepaired DNA.

Nucleotide metabolism is essential for DNA repair, providing the necessary building blocks to counteract damage, such as oxidative-induced 8-oxoguanine. Nucleotide imbalance can increase the frequency of somatic mutations [13, 14]. Neurons rely primarily on the salvage pathway to maintain nucleotide pools for DNA repair. The rate-limiting step of this pathway involves the phosphorylation of deoxyribonucleosides by deoxyribonucleoside kinases [15]. In *Drosophila*, a multisubstrate deoxyribonucleoside kinase (dNK) facilitates this process, whereas in humans, four kinases, including deoxycytidine kinase (DCK), thymidine kinase 1 (TK1), thymidine kinase 2 (TK2), and deoxyguanosine kinase (DGUOK), carry out nucleotide phosphorylation [16]. dCK phosphorylates deoxycytidine, deoxyadenosine, and deoxyguanosine, whereas TK1 acts in the cytoplasm during the S phase of the cell cycle. TK2 and DGUOK function in mitochondria, phosphorylating pyrimidine and purine deoxyribonucleosides, respectively [17].

Here, we showed that PARP1 levels are increased in the AD brain. We found that higher *PARP1* expression is linked to increased AD risk and more somatic mutations. Using a fly model of AD, we identified a metabolic signature in AD animals characterised by decreased levels of phosphorylated nucleotides. Enhancing nucleotide metabolism via dietary supplementation or genetically increasing levels of *dNK* protects against AD pathology in animals. Finally, we used Mendelian randomisation (MR) and showed that higher expression of human *DGUOK* is linked to a lower risk of developing AD. Our results suggest that enhancing nucleotide metabolism could improve DNA repair and serve as an adjunct therapy to delay AD progression.

## Results

### Higher PARP1 levels are linked to increased AD risk

**Fig. 1:**
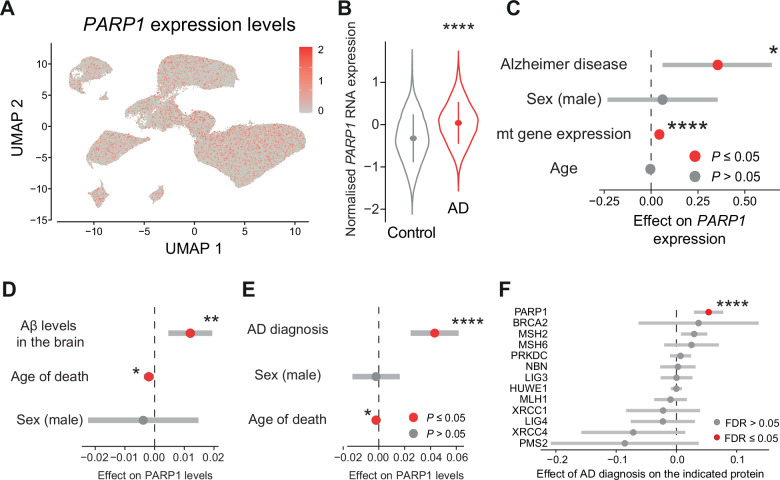
An AD diagnosis is linked to higher levels of *PARP* in the brain. **A** Analysis of *PARP1* expression levels in neurons from both patients with AD and controls. The cells are represented by dots, and the expression levels are represented in a two-colour heatmap, where red represents increased gene expression. **B**, **C** Excitatory neurons from patients with AD have increased expression of *PARP1* (**B**, asterisks, two-tailed Student’s *t*-test). This association remains consistent after accounting for age and sex as covariates (**C**, asterisks, *β*_*AD*_ = 0.35, standard error = 0.14, *P* = 0.02, linear mixed effect model). In (**B**), we show the distribution of the RNA expression levels of *PARP1* in patients with AD and healthy controls, where the circles represent the median and the vertical lines represent the interquartile ranges. **C** Statistical analysis of *PARP1* expression, accounting for age, sex, mitochondrial (mt) gene expression and patient ID as random effects. **D** Aβ and PARP1 protein levels in the brains of patients with AD are positively correlated (asterisks, *β*_*Aβ levels in the brain*_ = 0.012, standard error = 0.0038, *P* = 0.002, linear regression). The circles represent the effect size, and the horizontal lines represent the 95% confidence intervals. **E** An AD diagnosis is correlated with a higher level of PARP1 (asterisks, *β*_*AD diagnosis*_ = 0.043, standard error = 0.0095, *P* < 0.0001, linear regression). The circles represent the effect size, and the horizontal lines represent the 95% confidence intervals. **F** Association between DNA repair protein levels and AD diagnosis. Effect sizes and 95% confidence intervals from linear models testing the association between protein abundance and AD diagnosis (vs. control) for DNA repair proteins across four major pathways, including base excision repair (PARP1, XRCC1, and LIG3); mismatch repair (MSH2, MSH6, MLH1 and PMS2); homologous recombination (BRCA2 and NBN); and non‑homologous end joining (XRCC4, LIG4, PRKDC and HUWE1). Proteins with false discovery rate (FDR)–adjusted *P* ≤ 0.05 are indicated by red circles.

We previously reported that the expression of Aβ in flies increases Parp activity and decreases mitochondrial function. Furthermore, the genetic suppression of Parp in this fly model of AD improved mitochondrial function and was neuroprotective [7]. We also observed a significant increase in the levels of Parp in flies expressing Aβ (adjusted *P* = 0.021; controlled for false discovery rate using the Benjamini–Hochberg method [18]). Therefore, we tested whether there is an association between higher levels of PARP and AD. Using single-cell RNA sequencing of postmortem neurons from patients with AD [19], we detected *PARP1* in all clusters of excitatory neurons (Fig. 1A) and found higher levels of *PARP1* in neurons from patients with AD (Fig. 1B). This association remained consistent in a mixed effect model that accounts for several covariates (Fig. 1C). Next, using a large-scale proteomics dataset from AD brains [20], we found that patients with higher levels of Aβ in the brain (Fig. 1D) and an AD diagnosis (Fig. 1E) also had higher levels of the PARP1 protein. To conduct a comprehensive analysis of DNA repair pathways in AD brains, we analysed the the abundance of DNA repair proteins across four pathways, including PARP1, XRCC1, and LIG3 in base excision repair; MSH2, MSH6, MLH1 and PMS2 in mismatch repair; BRCA2 and NBN in homologous recombination; and XRCC4, LIG4, PRKDC, together with the E3 ubiquitin ligase HUWE1, which regulates PRKDC activity in non‑homologous end joining. We found that only PARP1 is significantly increased in AD brains (Fig. 1F). Taken together, these results support our use of a fly AD model [7] and show that PARP1 expression is increased in AD at both the mRNA protein level.

**Fig. 2:**
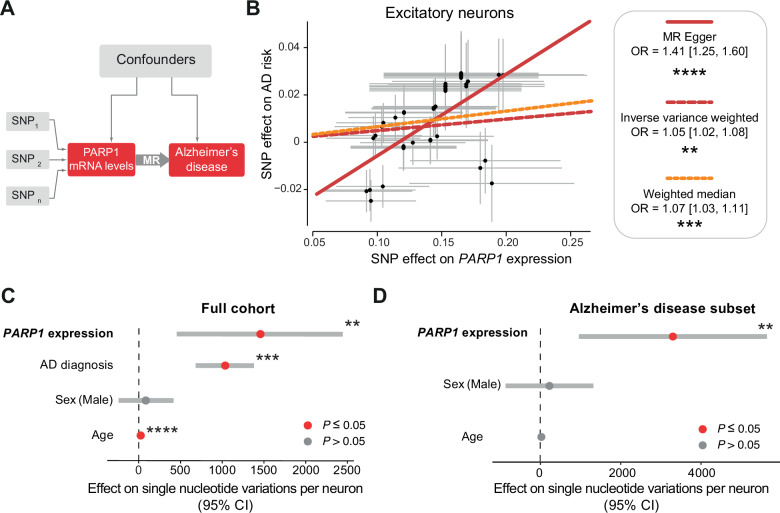
Higher predicted *PARP1* levels are associated with increased AD risk and more somatic mutations in neurons. **A** Schematic illustration of Mendelian randomisation (MR). MR uses genetic variants (SNPs in grey) that are associated with an exposure (*PARP1* mRNA levels in red) as instrumental variables to evaluate the causal effect (large grey arrow) of that exposure on an outcome (Alzheimer’s disease risk in red). Confounders are represented by the large grey box. **B** Increased *PARP1* expression in excitatory neurons increases AD risk. The statistical methods that were used and their significance are indicated. **C**, **D**. The predicted levels of *PARP1* in the brain were associated with increased single-nucleotide variations in the whole cohort (**C**, β = 1455.60, standard error = 489.87, *P* = 0.006, linear mixed effect model) and in Alzheimer’s disease subset patients (**D**, β = 3299.00, standard error = 1179.05, *P* = 0.007, linear mixed effect model).

Next, we investigated the impact of higher *PARP1* expression on the risk of developing AD using MR. MR is less prone to bias and confounding factors than observational studies are and can provide more robust evidence for inferring causality [21]. We obtained germline genetic variants (single-nucleotide polymorphisms, SNPs) associated with PARP1 expression levels to examine their causal effect on AD (Fig. 2A). By combining several statistical methods [21] to assess the effect of SNPs associated with *PARP1* expression in excitatory neurons [22] and AD risk [23], we found that higher *PARP1* expression predicted an increased risk of developing AD (Fig. 2B). Taken together, these results indicate that the levels of PARP1 are greater in patients with AD than in controls and that higher *PARP1* expression is associated with an increased risk of developing AD.

### Higher *PARP1* levels are associated with more somatic mutations in AD

DNA damage causes somatic DNA mutations that accumulate during ageing and AD [3, 24], and PARP1 functions in DNA repair to mitigate this damage [25]. We next investigated whether *PARP1* expression levels were associated with a greater number of somatic mutations in human neurons. We first predicted the expression of *PARP1* in the brain based on SNPs [26] and then investigated how this predicted expression correlated with the number of sSNVs in single neurons [3]. We found that higher predicted *PARP1* expression was associated with more DNA mutations in neurons (Fig. 2C). Because PARP1 is activated during the DNA damage response [4, 25] and patients with AD have increased numbers of sSNVs in neurons [3], we next investigated the effect of increased *PARP1* expression in patients with AD. We found that higher *PARP1* expression was correlated with an increased number of somatic mutations in patients with AD (Fig. 2D), and the effect size was greater than that of the whole cohort. Taken together, these results indicate that *PARP1* is elevated in AD, increases AD risk and contributes to more somatic mutations.

### Higher neuronal expression of nucleotide salvage components protects against AD pathology

**Fig. 3:**
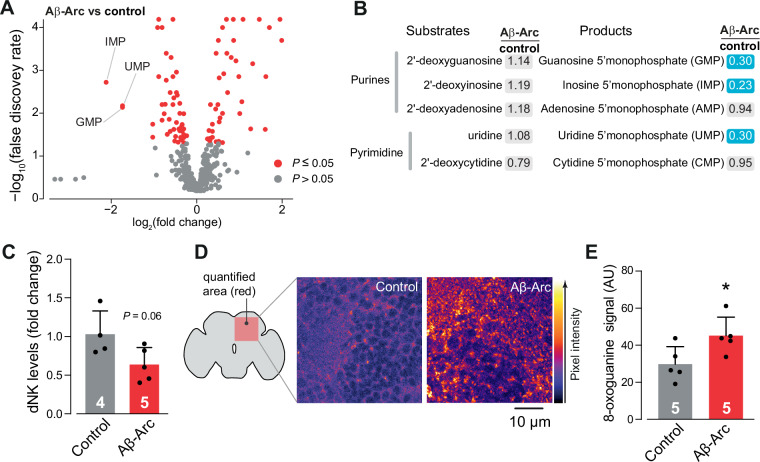
Aβ-Arc expression in flies decreases nucleotide levels. **A** Analysis of altered metabolites in Aβ-Arc-expressing flies. Metabolites that significantly changed are labelled in red (Welch two-sample *t*-test with Benjamini‒Hochberg correction for multiple comparisons). Nucleotide monophosphates are indicated. **B** Aβ-Arc expression causes decreases in nucleotide levels. Blue corresponds to metabolites that are significantly downregulated, and nonsignificantly altered metabolites are in grey. **C** Deoxynucleoside kinase (dNK) expression is decreased in the heads of flies expressing Aβ-Arc (mean ± s.d.; asterisks, two-tailed Student’s *t*-test). **D, E** Flies expressing Aβ-Arc have higher 8-oxoguanine levels in the brain than controls do (**E**, mean ± s.d.; asterisks, two-tailed Student’s *t*-test).

The increased activity of PARP enzymes can compromise neuronal health by depleting cellular stores of ATP and NAD^+^ [7]. Suppressing Parp in a fly model of AD increased NAD^+^ levels and improved lifespan [7]. Although PARP1 is involved in repairing DNA breaks following damage, higher PARP1 expression was linked to a greater number of SNVs (Fig. 2C, D). We hypothesised that a lower nucleotide availability in AD could account for an increase in the error rate of DNA repair processes. We tested this hypothesis in a fly model of AD by comparing the metabolomes of control flies and flies expressing Aβ-Arc. We found that the levels of guanosine 5′ monophosphate (GMP), inosine 5′ monophosphate (IMP) and uridine 5′ monophosphate (UMP) were the most significantly decreased metabolites in our analysis (Fig. 3A).

In the nucleotide salvage pathway, nucleoside and nucleotide kinases directly phosphorylate deoxyribonucleosides to their corresponding phosphorylated forms [15, 17]. We compared the levels of deoxynucleotides and their phosphorylated versions and found that although multiple phosphorylated nucleotides were decreased, the levels of unphosphorylated nucleotides were not significantly different (Fig. 3B). In flies, a single kinase, dNK, phosphorylates deoxynucleosides [27, 28]. Because we did not detect dNK in our proteomics analysis of Aβ-expressing flies, we measured its mRNA levels and found that these flies presented lower levels of dNK transcripts in the head (Fig. 3C).

Age and a diagnosis of AD are associated with increased levels of somatic mutations, as well as DNA damage in the form of oxidised guanine or 8-oxoguanine [3, 29, 30]. 8-Oxoguanine is a major mutagenic base lesion caused by oxidative damage [31]. It is possible that, in AD, oxidative stress damages the guanine nucleotide in the DNA, which neurons attempt to repair, thus depleting the nucleotide pool. Because we observed an altered nucleotide pool in patients with AD as well as signs of somatic mutations in patient data, we quantified the levels of 8-oxoguanine in the fly brain. We found that Aβ-expressing flies had higher levels of 8-oxoguanine than controls (Fig. 3D, E), corroborating previous results in human neurons [3]. These results suggest that nucleotide metabolism is impaired in our AD model and that increasing dNK levels might confer protective effects.

**Fig. 4:**
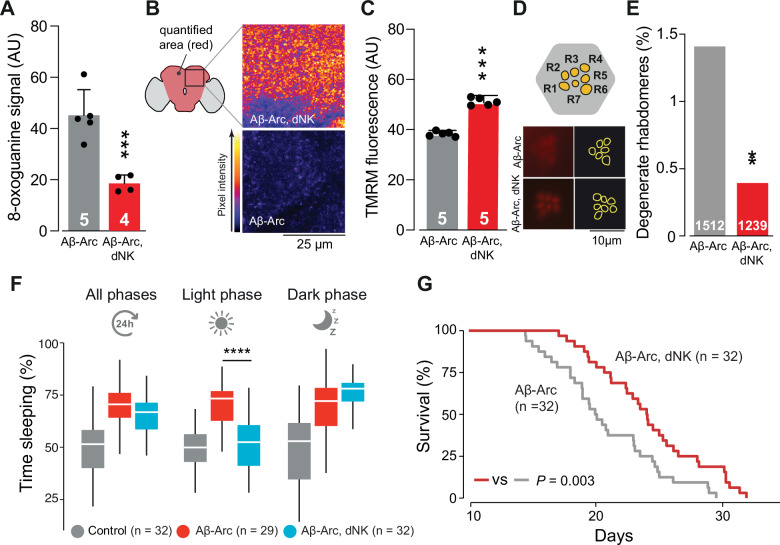
Deoxyribonucleoside kinase overexpression in flies confers protection against Aβ toxicity. **A**–**E** *dNK* overexpression in flies expressing Aβ-Arc decreases the accumulation of oxidised guanosine (**A**, asterisks, mean ± s.d., one-way ANOVA with Dunnett’s multiple comparison test), improves the Δψm in the brain (**B** and **C**, means ± s.d.; asterisks, two-tailed unpaired *t*-test) and reduces the neurodegeneration of photoreceptor cells (**D** and **E**, asterisks, two-tailed chi-square test, 95% CI). The dataset labelled Aβ-Arc in (**A**) is also used in Fig. 3C, as data from these genotypes were obtained as a single experimental set before statistical analysis. **F** The increase in light-phase sleep duration in Aβ-Arc-expressing flies was suppressed by the overexpression of *dNK* (asterisks, two-tailed unpaired *t*-test with Benjamini‒Hochberg correction for multiple comparisons). **G** Overexpression of *dNK* in Aβ-Arc-expressing flies increases their lifespan (log-rank, Mantel–Cox test).

Therefore, we tested whether overexpressing *dNK* could alleviate Aβ-related neurodegeneration in a fly model of AD. The overexpression of *dNK* in flies overexpressing Aβ-Arc decreased 8-oxoguanine levels (Fig. 4A), increased the mitochondrial membrane potential (Δψm) in the fly brain (Fig. 4B, C) and was neuroprotective (Fig. 4D, E). We previously reported that flies expressing Aβ-Arc sleep longer than controls do, replicating observations that patients with AD sleep longer than non-AD individuals do [7, 32]. We thus measured sleep duration as a behavioural marker of healthspan and found that *dNK* expression increased wakefulness during the light phase of the sleep cycle (Fig. 4F), as did overall lifespan (Fig. 4G). Taken together, these results show that increasing *dNK* expression protects against AD pathology.

**Fig. 5:**
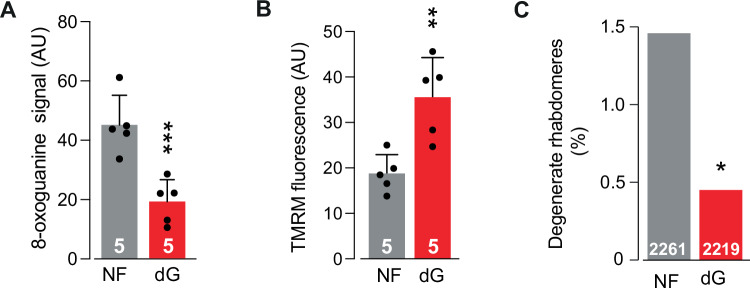
Dietary supplementation with deoxyguanosine is neuroprotective in flies expressing Aβ-Arc. **A**–**C** Supplementation with deoxyguanosine in Aβ-Arc-expressing flies decreases the accumulation of oxidised guanosine (**A**, asterisks, mean ± s.d., one-way ANOVA with Dunnett’s multiple comparison test), improves the Δψm in the brains of Aβ-Arc-expressing flies (**B**, means ± SDs; asterisks, two-tailed unpaired *t*-test) and reduces the neurodegeneration of photoreceptor cells in Aβ-Arc-expressing flies (**C**, asterisks, two-tailed chi-square test, 95% CI). The dataset labelled NF in (**A**) is also used in Figs. 3C and 5A, as data from these genotypes were obtained as a single experimental set before statistical analysis.

Next, we investigated whether supplementing the diet of Aβ-Arc-expressing flies with a nucleotide precursor could alleviate neurodegeneration in this model. Two purines, IMP and GMP, were most significantly decreased in Aβ-Arc-expressing flies (Fig. 3A, B). GMP can be synthesised from IMP in two steps [33]. In the first step, the hypoxanthine base is oxidised by IMP dehydrogenase to produce the base xanthine and the nucleotide xanthosine monophosphate (XMP). Glutamine then donates the amide nitrogen to XMP to form GMP in a reaction that is catalysed by GMP synthetase. Given the decrease in the guanosine pool, we hypothesised that supplementing the food of Aβ-Arc-expressing flies with deoxyguanosine could be neuroprotective. We found that dietary supplementation with deoxyguanosine alleviated 8-oxoguanine levels (Fig. 5A), improved the Δψm (Fig. 5B) and decreased neurodegeneration (Fig. 5C). Taken together, these results suggest that enhancing nucleotide metabolism by upregulating *dNK* or a diet supplemented with deoxyguanosine delays neurodegeneration in an animal model of AD.

**Fig. 6:**
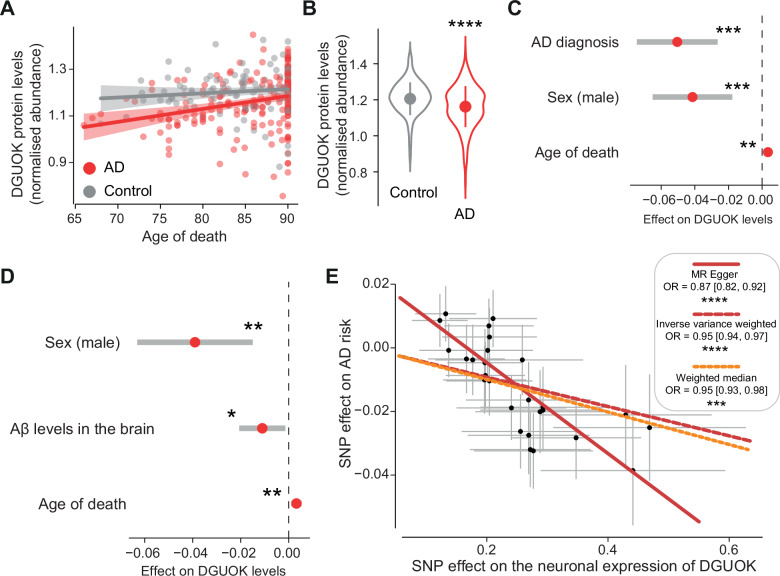
Higher levels of *DGUOK* decrease AD risk in patients. **A** Relationships between AD diagnosis, DGUOK protein level and age at death. Linear regressions (lines) are shown, with the shaded areas corresponding to the standard errors. **B** Patients with an AD diagnosis have lower DGUOK protein levels (asterisks, mean ± s.d., Welch two-sample *t*-test). **C** The relationship between DGUOK level and AD diagnosis remained significant after accounting for multiple covariates (asterisks, *β*_*AD diagnosis*_ = −0.050, standard error = 0.012, *P* < 0.0001, linear regression). **D** Aβ levels in the brain are negatively correlated with DGUOK levels (asterisks, *β*_*Aβ levels in the brain*_ = −0.011, standard error = 0.0049, *P* = 0.03, linear regression). **E** Mendelian randomisation shows that higher *DGUOK* expression in neurons decreases AD risk. The statistical methods and significance are indicated in the figure.

To explore the relevance of nucleotide metabolism in humans, we next examined whether components of the pathway are altered in the brains of patients with AD. Flies rely on a single, broad-specificity deoxynucleoside kinase (dNK) for nucleotide salvage, and we found that either upregulating *dNK* or supplementing the flies’ diet with nucleotide precursors was neuroprotective in both an AD model (Figs. 3 and 4) and a Parkinson’s disease model [34, 35]. In contrast, nucleotide salvage in humans involves four specialised deoxynucleoside kinases: DGUOK, TK1, TK2, and dCK. We focused on DGUOK because it specifically phosphorylates deoxyguanosine within mitochondria [36], a site of high oxidative stress and DNA damage in AD. Thus, we sought to examine whether DGUOK expression is altered in human AD brains and whether it may play a role in disease pathology. By assessing the protein levels of DGUOK in 309 patients [20], we found that DGUOK levels were decreased in patients with AD (Fig. 6A–C). Because we used a *Drosophila* model of AD expressing Aβ, we investigated the correlation between Aβ levels in the brain and DGUOK levels. We found that higher Aβ levels were associated with a lower level of DGUOK (Fig. 6D), supporting our findings in a fly model of AD. We next sought to test the causal effects of higher DGUOK expression on AD risk. Using MR to model the relationship between DGUOK and AD risk, we found that higher DGUOK expression predicted a lower risk of developing AD (Fig. 6E). Taken together, our results show that genetically enhancing nucleotide metabolism is protective in a fly model of AD, which is supported by data from patients with AD.

## Discussion

Postmortem brains from patients with AD show markers of DNA damage in the form of somatic mutations [3, 37] and the activation of DNA repair proteins [6]. We detected increased levels of PARP1 in the brains of patients with AD. Although PARP1 is involved in DNA repair, we found that higher PARP1 levels were associated with more somatic mutations. In cancer, decreased nucleotide bioavailability increases tumorigenicity [13]. Using a fly model of AD, we found that the levels of nucleotides such as guanosine monophosphate were the most significantly decreased metabolites. We show that enhancing nucleotide bioavailability in animals delays AD pathology and that a similar gene in humans also predicts AD risk.

A prominent theory of ageing proposed that an age-related increase in somatic mutations is associated with a loss of information [38]. These somatic mutations can occur in key genes linked to AD pathology and increase the risk of developing AD [39], which could lead to the production of immunogenic peptides [40] or cause protein misfolding [41]. Although a higher rate of somatic mutations, modelled via mutations in DNA polymerases ε and δ, does not increase age-related phenotypes [42], deficiency in DNA damage repair mechanisms exacerbates cognitive decline in AD models [43, 44].

In line with these observations, we observed increases of the protein and mRNA levels of PARP1. However, we found that higher predicted *PARP1* mRNA expression levels were correlated with increased somatic mutations in AD. These findings suggest that the DNA repair mechanism may introduce somatic mutations. Specifically, our observations are: first, individuals with higher *PARP1* expression, based on germline variants fixed at conception, have more somatic mutations in neurons (Fig. 2), indicating a potential causal relationship; second, we observed an imbalance in nucleotide pool in our *Drosophila* AD model (Fig. 3A, B); finally, nucleotide insufficiency is known to promote replication stress and genomic instability, which can be rescued by exogenous nucleosides [13]. Therefore, we reason that oxidative stress in AD increases DNA damage and upregulates *PARP1*, but during repair, limited nucleotide availability might lead to errors and somatic mutations. Without efficient repair, cells would undergo apoptosis or senescence rather than accumulating mutations. The links between *PARP1* expression, nucleotide availability, and somatic mutations require additional causal investigations and direct measurements of mutation burden using sequencing methods, which we did not perform in our study.

It is conceivable that reducing the burden of somatic mutations in the brain might serve as a neuroprotective approach to mitigate or delay AD. A few strategies, including removing highly mutated cells, reprogramming the epigenome to enable DNA repair or correcting these mutations through CRISPR/dCas, have been proposed [45], but none of these methods seem to be plausible for AD treatment. Using metabolomics, we found that the levels of GMP were substantially decreased in our fly model of AD and that enhancing nucleotide metabolism could delay AD pathology. Based on evidence from cancer research on the importance of maintaining nucleotide bioavailability for genome stability [46, 47], enhancing nucleotide metabolism could delay AD pathology through the maintenance of genome stability. It is thus possible that, in cases of oxidative DNA damage such as in AD, an increased DNA damage response disrupted the bioavailability of guanosines, which caused errors in the repair process. A limitation of our study was the measurement of GMP and not deoxyguanosine monophosphate (dGMP), the substrate for DNA repair or replication [48, 49]. dGMP levels were not detected in our analysis. Future research could use LC‒MS to measure dGMP levels in patient samples and animal models of AD to verify our results.

In flies expressing Aβ-Arc, we observed decreased levels of phosphorylated ribonucleotides, increased levels of markers of DNA damage and decreased expression of *dNK*, suggesting that the activity of dNK might be decreased. We attempted to measure dNK protein levels through mass spectrometry in our fly models, but were unable to detect this enzyme. We showed that deoxyguanosine supplementation and *dNK* overexpression delayed AD pathology in a fly model. However, we did not quantify the accumulation of somatic mutations in the fly brain. We measured levels of 8-oxoguanine, which is a marker of oxidative DNA damage, and observed that enhancing nucleotide metabolism reduced 8-oxoguanine, suggesting improved DNA repair. However, we acknowledge that the beneficial effect of deoxyguanosine supplementation on 8-oxoguanine levels could be mediated indirectly through improved redox homeostasis, rather than a direct consequence of restored nucleotide pools. Direct measurements of the accumulation of somatic mutations in the brains of animal models of AD using bulk or single-cell genomics approaches would be needed to confirm our conclusions on the effect of nucleotide metabolism on the rate of somatic mutations.

There could also be additional mechanisms by which manipulating nucleotide metabolism could delay AD pathology, independent of somatic mutations. For example, enhancing nucleotide metabolism could increase replication of the mitochondrial genome and mitochondrial biogenesis. We previously showed that enhancing nucleotide metabolism is neuroprotective in animal and cell models of Parkinson’s disease, possibly through increasing the mitochondrial DNA copy number [34]. Further investigations into markers of DNA damage in the form of either 8-oxoguanine or somatic mutations upon manipulation of nucleotide metabolism in the context of oxidative stress are also important for improving the efficiency of nucleotide supplementation. For example, previous research has shown that cells can repair DNA with oxidatively damaged nucleotides [50, 51]. Therefore, supplementing cells with deoxyguanosine in conjunction with antioxidants could be important in promoting their efficiency in preventing somatic mutations. Similarly, we have not performed any longitudinal tracking of the neurodegeneration or lifespan in models of AD expressing Aβ-Arc or hyperphosphorylated tau supplemented with deoxyguanosine or overexpressing *dNK*. Future research could investigate nucleotide metabolism at both the genetic and metabolomic levels in rodent models of AD or in patients with AD, test whether these markers correlate with somatic mutations, and assess whether there could be any synergistic effects with antioxidant supplementation.

Our work indicates that *PARP1* expression is correlated with the number of somatic mutations in the brain. Future work could address transcriptome-wide associations [52] of specific genes linked to the number of somatic mutations in neurons. However, this would be linked to the strength of any association between an SNP and the expression of a target gene. We used published data to predict the expression of *PARP1* in a cell type-specific manner [22] but did not identify enough eQTLs to make an accurate prediction for *DGUOK*. Future research can leverage the increasing number of single-cell transcriptomes in AD [53], eQTL data and information on somatic mutations to predict genes that may increase or delay the rate of somatic mutations. Elucidating the genetic mechanisms that moderate the accumulation of somatic mutations could be critical in maintaining genome integrity and preventing cancer and age-related diseases such as AD.

## Methods

### Genetics and *Drosophila* strains

Fly stocks and crosses were maintained on standard cornmeal agar medium at 25 °C. The strains used were *elavGAL4*, *w; UAS Aβ42ARC;* + and *w; 51D;* + (described in [7]), and *w; UAS_dNK;* + (described in [34]).

### Metabolic profiling

The metabolites were analysed by global metabolic profiling using the Metabolon Platform (Metabolon Inc., NC, USA) as previously reported [7, 32].

### Drug treatments

Deoxyguanosine (dG) was incorporated directly into the fly food at a final concentration of 0.5 mg/ml. The adult flies were kept on drug-containing food from eclosion, and they were transferred to vials with fresh food every 3 days. Flies from each genotype were randomly assigned to normal food or supplemented food.

### Locomotor assays and lifespan analysis

The analyses of sleep and lifespan in flies were performed as described previously [7, 18, 32, 54]. Adult male flies were aged to 10 days posteclosion and individually loaded in glass tubes containing the same food used for rearing. The flies were grown and analysed under a light/dark cycle of 12 h/12 h at 25 °C. The total number of recorded midline crossings per minute was recorded using a *Drosophila* Activity Monitoring System (Trikinetics, Waltham, MA), and the data were analysed using Rethomics. The analysis started at the first ZT0 to allow acclimation. Sleep duration was calculated for the first 5 days, and the data for the flies that died were discarded. Sleep was defined as 5 min of inactivity. One-way analysis of variance with Tukey’s multiple comparison test was used to determine the significance of the fraction of time asleep. The data for lifespan analysis are presented as Kaplan–Meier survival distributions. We recorded the entire lifespan of the flies from 10 days posteclosion until death and determined their statistical significance using the log-rank test. The full analysis script and the raw data are available in our GitHub repository: http://izu0421.github.io/abeta_mutations.

### Microscopy-based assessment of mitochondrial membrane potential and 8-oxoguanine

Measurements of the Δψm in fly brains were performed as previously described [18]. Briefly, fly brains were incubated for 20 min at room temperature with 40 nM TMRM in Hank’s Balanced Salt Solution (14175095, Gibco), and the dye was added during the experiment. In these experiments, TMRM was used in redistribution mode to assess the Δψm; therefore, a reduction in TMRM fluorescence represented mitochondrial depolarisation. Confocal images were obtained using a Zeiss LSM 880 confocal microscope. The illumination intensity was maintained at a minimum (0.1–0.2% of the laser output) to avoid phototoxicity, and the pinhole was set to give an optical slice of 2 μm. The fluorescence was quantified by exciting TMRM with a 565 nm laser and measuring above 580 nm. Z-stacks were acquired, and the mean maximal fluorescence intensity was measured for each group. The fluorescence of the entire midbrain was quantified.

For the measurement of 8-oxoguanine levels, fly brains dissected in cold PBS were fixed in Carnoy solution (ethanol:acetic acid = 3:1) at 4 °C overnight and washed with 2 M HCl for 2 min. Samples were then washed three times with PBS, 0.1% Triton for 10 min at room temperature and blocked with 0.5% Triton, 10% goat serum in PBS at 4 °C overnight. Anti-8-oxo-dG antibody (ab206461, Abcam) was added to the samples at 1:50 of the blocking buffer and incubated at 4 °C overnight and washed thrice with PBS, 0.1% Triton for 10 min at room temperature. The AlexaFluor 488 goat anti-mouse IgG secondary antibody (A-11001, Invitrogen) was used at 1:250 in PBS, 0.1% Triton, incubated for 2 h at room temperature, and washed three times with PBS, 0.1% Triton for 10 min at room temperature, with the middle wash containing Hoescht at 1:500 (33342, Invitrogen). Confocal images were obtained using a Zeiss LSM 880 confocal microscope with a 63× objective.

### Pseudopupil analysis

The heads of 10-day-old flies were directly fixed on standard microscope slides using quick-dry transparent nail varnish as described previously [7, 55]. A Zeiss Axioplan 2 microscope equipped with a ×63 oil immersion objective was used to visualise the ommatidia. In all, 5 flies per condition were examined. The percentage of abnormal rhabdomeres was calculated as the number of degenerate rhabdomeres over the total number of rhabdomeres: (A × 1 + B × 2 + C × 3)/N, where A is the number of ommatidia with 6 rhabdomeres, B is the number of ommatidia with 5 rhabdomeres, C is the number of ommatidia with 4 rhabdomeres and N is the total number of ommatidia counted. Statistical significance was determined using the chi-square test.

### RNA extraction and quantitative real-time PCR with reverse transcription

Total RNA was extracted from 30 heads per sample gut using TRIzol (Ambion) and quantified by spectrophotometric analysis (Nanodrop, Thermo Scientific). Quantitative real-time PCR with reverse transcription (RT–qPCR) was performed with a real-time cycler (Applied Biosystems 7500, Fast Real-Time PCR Systems) using a SensiFAST SYBR Lo-ROX One-Step Kit (Bioline). The fold change values were calculated using the comparative Ct method [56]. For RT–qPCR, we measured the coefficient of variation (CV) of the technical replicates and excluded any samples with a CV > 3% from the statistical analysis as exemplified previously [57]. The dNK primer set was obtained from QIAGEN (QuantiTect Primer Assays, no. QT0097774). rp49 was used as a housekeeping gene: forwards, 5′-TGTCCTTCCAGCTTCAAGATGACCATC-3′; reverse, 5′-CTTGGGCTTGCGCCATTTGTG-3′.

### Two-sample Mendelian randomisation

For two-sample Mendelian randomisation, we followed the STROBE-MR (strengthening the reporting of observational studies in epidemiology using Mendelian randomisation) guidelines where appropriate [58]. We used a combination of 3 statistical methods (inverse variance weighted, weighted median and Egger regression) for robustness using the TwoSampleMR package [59] in R. A genome-wide association study of AD was obtained from a recently published GWAS on AD [23]. This dataset was chosen because it contains GWAS data on AD with the largest sample size. For the cell type-specific Mendelian randomisation analysis, we incorporated the exposure data of cell-specific expression quantitative trait loci (eQTLs) [22]. We only considered statistically significant eQTLs linked to the expression of genes of interest in neurons (nominal significance of *P* < 0.05). We performed clumping to remove correlated SNPs using the 1000 Genomes EUR reference panel. A high threshold of an r^2^ value of 0.95 and a distance within 100 kb was used to increase the number of SNPs available per gene, which in turn improved the ability to detect an effect. When clumping at r^2^ = 0.95, the SNPs were not strictly independent. This potential issue was remediated using 3 independent statistical tests, as well as experimental validation in a model organism. Multiple testing was not performed, as this study investigated the link between a single gene and AD risk per analysis using MR. The forest plots were generated using yytools (https://github.com/izu0421/yytools). Additional analyses are available via the GitHub repository of this study: http://izu0421.github.io/abeta_mutations.

### Analysis of single-cell data

For the analysis of gene expression in single-cell RNA-seq data, the processed sequence reads from a previous study [19] were analysed using scanpy in Python 3.7. The data were preprocessed according to best practices for single-cell RNA sequencing analyses [60, 61], with the removal of cells with less than 10 genes expressed and cells with a mitochondrial gene ratio above 20%. Gene expression was then log_2_-normalised. For the specific differential expression analyses of each gene of interest, only cells that expressed that gene were selected for downstream analysis. Next, we used linear regression on single neurons to query the associations between the expression levels of the genes and their AD status, accounting for age and sex. We performed additional analyses using linear mixed models that account for interindividual differences in gene expression and added more covariates, such as mitochondrial gene expression levels and cell subtypes.

### Analysis of protein levels from mass spectrometry data

For the analysis of the protein levels of PARP1 and DGUOK in the brains of patients with AD, we used a large dataset from a previous publication [20], following our previous workflows [62]. The batch-corrected and normalised data from controls and symptomatic patients with AD were used for subsequent analysis and are available here: https://www.synapse.org/DeepConsensus. For statistical analyses, we used linear regression models to investigate the effect of our variable of interest (i.e., Aβ levels or AD diagnosis) with covariates (sex and age of death) on the levels of each protein of interest in the brain.

### Inference of the effect of *PARP1* expression on somatic mutations

We estimated genetically predicted expression levels of *PARP1* using eQTLs of human brain tissue reference panels from GTEx [26]. Somatic mutation data were obtained from previously published single-neuron whole-genome sequencing datasets, which reported counts of SNVs in individual neurons from postmortem human brain samples generated via multiple displacement amplification [3]. We tested whether predicted *PARP1* expression was associated with somatic mutation burden using a linear mixed effect model and the maximum likelihood method, and *P* values from a *t*-test with the Satterthwaite approximation were calculated for each fixed effect as implemented in the lmerTest. Age, sex, and diagnosis were included in the models where applicable. Data from all donors were used, which consisted of 159 neurons from 21 control donors and 91 neurons from 8 donors with AD.

### Statistical analyses

Statistical analyses were performed using R version 4.0.2 and GraphPad Prism (www.graphpad.com). The data are presented as the mean values, and the error bars indicate ± the SDs. The number of biological replicates per experimental variable (*n*) is indicated in either the respective figure or the figure legend. No samples were excluded from the analysis unless otherwise stated. Blinding was not performed. Significance is indicated as * for *P* ≤ 0.05, ** for *P* ≤ 0.01, *** for *P* ≤ 0.001, **** for *P* ≤ 0.0001 and NS for *P* > 0.05.

### Digital image processing

The fluorescence images were acquired as uncompressed bitmapped digital data and processed using FIJI with established scientific imaging workflows. To visualise pixel intensity, confocal images acquired with identical settings were processed using a heatmap.

## Data Availability

All data for are available in GitHub (http://izu0421.github.io/abeta_mutations). All other data are available upon reasonable request.

## Code availability

All the code is available in our GitHub repository (http://izu0421.github.io/abeta_mutations).
